# Clinical and molecular characteristics of congenital hypothyroidism with *DUOX2* mutations

**DOI:** 10.1186/1687-9856-2013-S1-O54

**Published:** 2013-10-03

**Authors:** Yoo-Mi Kim, Hye Young Jin, Beom Hee Lee, Sun-Hee Heo, Gu-Hwan Kim, Han-Wook Yoo

**Affiliations:** 1 Department of Pediatrics, Asan Medical Center Children’s Hospital, University of Ulsan College of Medicine, Seoul, Korea; 2 Genome Research Center for Birth Defects and Genetic Disorders, Asan Medical Center Children’s Hospital, University of Ulsan College of Medicine, Seoul, Korea; 3 Medical Genetics Center, Asan Medical Center Children’s Hospital, University of Ulsan College of Medicine, Seoul, Korea

## Aims

Biallelic or monoallelic mutations in *DUOX2* have caused congenital hypothyroidism (CH) with variable phenotypes from asymptomatic to permanent CH. This study was aimed to clarify molecular feature and clinical spectrum in CH with *DUOX2* mutations.

## Methods

This study included 62 transient or permanent CH patients with normal-sized or enlarged eutopic thyroid. All coding exons of *DUOX2* and their intronic flanking sequences were amplified by PCR, and directly sequenced. As for novel sequence variant of *DUOX2*, functional studies were performed by measuring H_2_O_2_ generation *in vitro*. Clinical presentation was retrospectively reviewed based on medical records.

## Results

Fifteen different *DUOX2* variants including 11 novel variants (p.N43Y, p.A72S, p.P96L, p.G206V, p.V779M, p.A1123T, p.Y1229C, p.R1334W, p.C1411Y, p.I1417F, p.Q202RfsX93) were identified in 21 of 62 patients, indicating 33.9% of prevalence. Functional single nucleotide polymorphism (SNP), p.H678R, was more frequently found in CH patients than in healthy individuals (allele frequency: 12.9 % vs. 5.5 %, *P*=0.023). *DUOX2* variants were observed 11 out of 28 transient CH patients and 10 out of 32 permanent CH patients (39.3 % vs 31.3 %, *P*=0.593). At reevaluation, thyroid stimulating hormone (TSH) levels were 7.4±1.9 mU/L and 27.4±21.2 mU/L in transient and permanent CH with *DUOX2* variants, respectively. Five patients with biallelic variants had higher initial TSH level (116.1±79.2 vs 50.2±50.2, *P*=0.049), and 3 out of 5 were determined as transient CH. Functional analysis revealed partially impaired H_2_O_2_ generation in 15 different DUOX2 mutants.

## Conclusion

This study showed that *DUOX2* mutation is a common cause of CH with normal-sized or enlarged eutopic thyroid. Clinical spectrum of *DUOX2* mutations was variable, emphasizing the importance of alternative mechanism to compensate the function of DUOX2 or modifying factors to regulate DUOX2 expression. Long-term follow up for CH patients with *DUOX2* variants should be needed.

